# A Randomized Control Study to Evaluate the Efficacy of Safe Home Toolkit for Under-Five Children (SHT-UFC) on the Awareness of Parents Regarding the Prevention of Domestic Accidents

**DOI:** 10.7759/cureus.31416

**Published:** 2022-11-12

**Authors:** Usha Shende, Jayant D Vagha, Archana Maurya

**Affiliations:** 1 Department of Child Health Nursing, Kasturba Nursing College, Sewagram, Wardha, IND; 2 Department of Pediatrics, Jawaharlal Nehru Medical College, Datta Meghe Institute of Medical Sciences, Wardha, IND; 3 Department of Child Health Nursing, Srimati Radhikabai Meghe Memorial College of Nursing, Datta Meghe Institute of Medical Sciences, Wardha, IND

**Keywords:** parents, safe home toolkit for under-five children, serious health issue, unintentional, childhood injuries

## Abstract

Background

Domestic accidents among under-five children are linked to numerous factors, including child development, the physical layout and facilities at home, knowledge and practices, the behavior of parents and other family members, parents' education, overcrowding and homelessness, and the availability of safety equipment in the home.

Objective

The objective of this study is to evaluate the efficacy of a safe home toolkit for under-five children (SHT-UFC) on parents' awareness regarding preventing domestic accidents among under-five children, to assess the baseline knowledge and practices regarding preventing domestic accidents among parents of under-five children in the control and experimental groups, to find out the reported incidence of domestic accidents among parents of under-five children in the control and experimental groups, and to evaluate the effectiveness of a safe home toolkit among parents of under-five children.

Method

Simple random sampling was done in each community to recruit the participants. The sampling frame (list) of the participants was collected from the Anganwadi workers of the community. Each participant was then selected by using the computer-generated table in both groups that are the experimental and control groups.

Result

Knowledge and practice scores regarding SHT-UFC are positively related in the pretest and post-test, indicating that knowledge influences practices. In the pretest, Pearson's correlation coefficient (r) is higher than in the post-test because the knowledge was less and so were the practices in the pretest. In the post-test, the knowledge gained is varied yet significant, and the practices also improved.

Conclusion

This study revealed that a safe home toolkit for under-five children (SHT-UFC) is effective in creating awareness among parents regarding the prevention of domestic accidents.

## Introduction

Accidental injuries in the home environment are the main reason for preventable deaths in under-five children. These injuries can also have negative consequences on a child's health and lead to serious disability. Children's accidental injuries remain a major public health concern [1]. Unintentional injuries (such as burn, fall, poisoning, cuts, and injuries from drowning and strangulation/choking/suffocation) are significant health issues that can be avoided among children. There is a persistent social dimension to accidental injuries. The rate of casualty visits for non-intentional injuries under five years is 38%, which is more common in poor populations than in affluent people [2].

Approximately 830000 children under 18 years die worldwide every year from unintentional injuries. Millions of children require hospitalization and advanced care every year for nonfatal injuries. Ninety-five percent of injuries to children occur in underdeveloped and developing countries. Forty percent of all child deaths in developed countries are due to child injuries [2]. Globally, nearly 47000 children and adolescents die due to falls each year. There are about 690 children who miss school due to fatal falls each year, and it is the main contributor to long-term disability in under-five children [3].

Choking, suffocation and strangulation, falls, poisoning, burns and scalds, and drowning are unintentional injuries accounting for 90% of hospitalization in this age group. These significant causes of preventable death and severe long-term harm demand urgent action to make a significant difference [3].

A serious bathwater burn would need years of severe skin grafts; therefore, treating an accident may be very costly. Permanent brain damage may be caused by a fall at home. Injuries significantly influence study, job, emotional health, and socialization. In addition, such long-term injuries are psychologically painful for the caregivers too. Unintentional injuries in the home accounted for almost 7% of all pediatric deaths in 2015 among children between one and four years of age [4-6].

Domestic accidents among under-five children are linked to numerous factors, including child development, the physical layout and facilities at home, knowledge, and practice, the behavior of parents and other family members, parents' education, overcrowding and homelessness, and the presence of safety equipment inside the house [7-9]. Domestic accident types have different levels of gravity. Some accidents, such as choking, strangulation, and drowning, could be fatal. Scalds and burns can cause serious long-term impairment and hospitalization but rarely result in death [10].

## Materials and methods

Research setting and design

In the selected community area of Wardha, Maharashtra, India, the study was carried out and used an experimental study approach. A randomized control study was the research method used for this study. The study proposes to evaluate the efficacy of a safe home toolkit for under-five children (SHT-UFC) among parents in preventing domestic accidents.

Sampling techniques, sample size, and eligibility criteria

Simple random sampling was done in each community to recruit the participants. The sampling frame (list) of the participants was collected from the Anganwadi workers of the community. Each participant was then selected by the computer-generated table for both groups that are experimental and control groups. In this study, 200 samples were included, 100 samples each from the experimental group and control group. The eligibility criteria for the participants are shown in Table 1.

**Table 1:** Sample Selection Criteria

Serial Number	Criteria for Participant Selection
	Inclusion criteria
1	Those who were available at the data collection gave consent to participate.
2	Parents of under-five children in the age group of 21-50 years.
3	Both male and female parents were included.
4	Subjects are selected from rural and urban communities.
5	Those who understand Hindi and Marathi.
	Exclusion criteria
1	Those parents of under-five children who were noncooperative and suffered from severe mental disorders.

Data collection tools

The approach to the study was experimental. The safe home toolkit intervention was designed to create awareness among the parents of children under five years. No interventional study was conducted with models of safe homes and videos on home safety for parents of under-five children. This age group is most vulnerable to domestic accidents. Hence, the study approach is an experimental study with a control group. The control group is used to ensure that the improvement in awareness is due to the intervention only. Simple random sampling is used to select and assign the participants. Every house has safety concerns and would have qualified for the study. However, the study was restricted to under-five children; hence, simple random sampling was used to avoid selection bias.

The material used for data collection

There was no standardized tool exactly matching the data collection needs for this study. However, guidelines by the WHO are available for safe homes for under-five children. The investigator prepared the knowledge questionnaire and safe home inventory based on those guidelines and validated them during the pilot study. The tools used for data collection were modified standardized tools. The tools used in this study include a demographic data sheet to collect information regarding the parent's age, gender, education, occupation, residence, monthly income, religion, type of house, number of under-five age group children, ownership of the house, etc. The researcher developed the knowledge questionnaire and validated it in the pilot study. There are 30 multiple-choice questions (MCQ) on different aspects of domestic accidents. The questions include the causes and preventive measures implemented at home to prevent a particular type of domestic accident. The correct option is scored with "1," and the wrong answer is scored with "0." The tool's reliability was estimated to be r=0.763 by Spearman-Brown correlation coefficient and r=0.762 by Guttman split-half coefficient. The tool was found reliable and hence used for primary study data collection.

Standardized general home safety inventory and intervention

This was developed from the WHO's guidelines for safe and healthy homes for children under five years. It includes observations in each housing area: hall, kitchen, bedrooms, garage, front courtyard, courtyard staircase, bathrooms, etc. The investigator makes the observations to estimate the level of the general safety of the house for under-five age group children. Each item is ticked on "yes" or "no." The presence of safety equipment or infrastructure got the score "yes" = 1, and the absence of such gadgets got the score "no" = 0. There is a total of 101 items in the inventory. This inventory was also validated during the pilot study and found reliable. The intervention was the safe home toolkit for under-five children (SHT-UFC). It consists of an information leaflet on home safety, including engineering measures, environmental measures, legislation and standards, development education and skills, videos on home safety, and a safe home model demonstration.

Implementation of SHT-UFC

On day 1, pretest data were collected from the participants. Then, they distributed an information handout regarding home safety. They were then shown the video on home safety. On day 2, they were asked to read the handout before the investigator and asked about any doubts. Their suspicions were clarified, and they were shown the ideal safe home model. They have explained the safety aspects of the model house. Handouts were left with the participants for easy reference.

Method of data collection and plan for data analysis

Following the Institutional Ethical Committee (IEC) of Datta Meghe Institute of Medical Sciences approval, the study was carried out. The parents' written informed consent was taken before recruiting them for the study. Permission from relevant stakeholders was taken. No harm of any nature is foreseen in the study. The data were collected on a one-on-one basis. The investigator prepared the sampling frame of families having under five years of age children in the selected community area. The families were chosen for recruiting in the study by computer-generated table. Only one parent from each family was included in the study. The baseline data were collected through self-reports (knowledge) and observations (practices). The interventions were given in groups of 5-10 parents. Overall data were collected for knowledge two times (baseline and seventh day of the intervention) and for practices three times (baseline, seventh day of the intervention, three months after the intervention, and after six months of intervention to assess the progress in practices). Data were analyzed using Statistical Package for Social Sciences (IBM SPSS Statistics, Armonk, NY) version 24.0.

Ethical aspects

The study proposal was presented to the Institutional Ethical Committee (letter number DMIMS(DU)/IEC/2018-19/7343). Prior permission is obtained from the sarpanch of the community to conduct the pilot study and the research study. Written informed consent is obtained from each study subject. The subjects are assured about the confidentiality of the information they provide. The data sheets are stored safely under lock and key with limited access to only the investigator and statistician. Random assignment of subjects was done to control and experimental groups. To prevent contamination, the control group was taken from a community geographically located at a distance from the experimental group. Both male and female parents were recruited for the study.

## Results

Distribution of participants according to their demographic characteristics

Table 2 shows the distribution of participants according to their demographic characteristics.

**Table 2:** Characteristics of Study Participants

Serial Number	Demographic Characteristics	Control Group		Experimental Group	
		Frequency	%	Frequency	%
1	Number of children in the age group 0-6 years				
	Nil	0	0	0	0
	1	73	73.0	58	58.0
	2	25	25.0	37	37.0
	3	1	1.0	4	4.0
	4	1	1.0	1	1.0
2	Type of house				
	Kaccha	16	16.0	20	20.0
	Pakka	84	84.0	80	80.0
3	Ownership of house				
	Owned	96	96.0	93	93.0
	Rented	4	4.0	7	7.0
4	Type of family				
	Nuclear	56	56.0	55	55.0
	Joint	44	44.0	45	45.0
5	Number of family members				
	2-4	41	41.0	48	48.0
	5-6	44	44.0	34	34.0
	7-8	7	7.0	13	13.0
	More than eight	8	8.0	5	5.0
6	Age of the father				
	21-30	27	27.0	24	24.0
	31-40	69	69.0	71	71.0
	41-50	4	4.0	5	5.0
7	Age of the mother				
	21-30	77	77.0	90	90.0
	31-40	23	23.0	9	9.0
	41-50	0	0	1	1.0
8	Education of the father				
	No formal education	2	2.0	0	0
	Primary	9	9.0	14	14.0
	Secondary	50	50.0	51	51.0
	Higher secondary	24	24.0	25	25.0
	Graduate	14	14.0	5	5.0
	Postgraduate	1	1.0	4	4.0
	Others	0	0	1	1.0
9	Education of the mother				
	No formal education	3	3.0	0	0
	Primary	2	2.0	10	10.0
	Secondary	44	44.0	35	35.0
	Higher secondary	29	29.0	40	40.0
	Graduate	15	15.0	12	12.0
	Postgraduate	7	7.0	2	2.0
	Others	0	0	0	0
10	Occupation of the father				
	Unemployed	0	0	0	0
	Self-employed	15	15.0	11	11.0
	Farmer	31	31.0	25	25.0
	Service	16	16.0	17	17.0
	Labor	38	38.0	47	47.0
11	Occupation of the mother				
	Homemaker	80	80.0	82	82.0
	Self-employed	1	1.0	2	2.0
	Farmer	2	2.0	0	0
	Service	4	4.0	3	3.0
	Laborer	13	13.0	13	13.0

Level of knowledge and practices during the pretest and post-test

Tables 3-4 display the distribution of study participants according to their level of knowledge regarding the safe home toolkit for under-five children before and after the intervention.

**Table 3:** Level of Knowledge Before the Intervention

Level of Knowledge Regarding Safe Home Before the Intervention	Score Range	Percentage Range	Control Group		Experimental Group	
			Frequency	%	Frequency	%
Poor	0-7	0-25	4	4.0	8	8.0
Average	8-15	26-50	22	22.0	42	42.0
Good	16-23	51-75	69	69.0	46	46.0
Excellent	24-30	76-100	5	5.0	4	4.0

**Table 4:** Level of Knowledge After the Intervention

Level of Knowledge Regarding Safe Home After the Intervention	Score Range	Percentage Range	Control Group		Experimental Group	
			Frequency	%	Frequency	%
Poor	0-7	0-25	0	0	0	0
Average	8-15	26-50	11	11.0	1	1.0
Good	16-23	51-75	75	75.0	2	2.0
Excellent	24-30	76-100	14	14.0	97	97.0

Tables 5-6 present the distribution of study participants according to their level of practice regarding the safe home toolkit for under-five children before and after the intervention.

**Table 5:** Level of Practices Regarding Safe Home Toolkit Before the Intervention

Level of Practices	Score Range	Percentage Range	Control Group		Experimental Group	
			Frequency	%	Frequency	%
Poor	0-24	0-25	60	60.0	72	72.0
Average	25-48	26-50	37	37.0	27	27.0
Good	49-71	51-75	2	2.0	1	1.0
Excellent	72-94	76-100	1	1.0	0	0

**Table 6:** Level of Practices Regarding Safe Home Toolkit After the Intervention

Level of Practices	Score Range	Percentage Range	Control Group		Experimental Group	
			Frequency	%	Frequency	%
Poor	0-24	0-25	40	40.0	0	0
Average	25-48	26-50	47	47.0	13	13.0
Good	49-71	51-75	12	12.0	22	22.0
Excellent	72-94	76-100	1	1.0	65	65.0

Tables 7-8 show the distribution of study participants according to the reported incidence of domestic accidents among children under five years of age before and after the intervention.

**Table 7:** Reported Incidence of Home Accidents Before the Intervention

Nature of Domestic Accidents	Control Group		Experimental Group	
	Frequency	%	Frequency	%
Falls	34	34.0	28	28.0
Cuts and other injuries	9	9.0	15	15.0
Poisoning	0	0	0	0
Burns	6	6.0	6	6.0
Drowning	0	0	0	0
Strangulation/choking/suffocation	3	3.0	3	3.0

**Table 8:** Reported Incidence of Home Accidents After the Intervention

Nature of Domestic Accidents	Control Group		Experimental Group	
	Frequency	%	Frequency	%
Falls	30	30.0	10	10.0
Cuts and other injuries	13	13.0	2	2.0
Poisoning	0	0	0	0
Burns	1	1.0	0	0
Drowning	1	1.0	0	0
Strangulation/choking/suffocation	2	2.0	0	0

Table 9 shows the significance level for the difference between the mean scores of knowledge among the study participants in the experimental group before and after the intervention.

**Table 9:** Level of Significance for Knowledge Regarding Safe Home in the Experimental Group S: significant

Test	Mean	SD	F-value	P-value
Pretest	14.89	5.19	20.756	0.001, S (p<0.05)
Post-test	26.72	1.90		

Table 10 shows the significance level for the difference between the mean scores of knowledge among the study participants in the control and experimental groups before the intervention.

**Table 10:** Level of Significance for Practices Regarding Safe Home Between Control and Experimental Group in Pretest S: significant

Group	Mean	SD	T-value	P-value
Control	17.55	4.53	3.977	0.001, S (p<0.05)
Experimental	14.89	5.19		

Table 11 shows the frequency distribution of incidences of common domestic accidents before and after the intervention in the experimental group.

**Table 11:** Comparison of Reported Incidence of Domestic Accidents Before and After the Intervention in the Experimental Group

Nature of Domestic Accidents	Pretest		Post-test	
	Frequency	%	Frequency	%
Falls	28	28.0	10	10.0
Cuts and other injuries	15	15.0	2	2.0
Poisoning	0	0	0	0
Burns	6	6.0	0	0
Drowning	0	0	0	0
Strangulation/choking/suffocation	3	3.0	0	0

Table 12 shows the difference between the mean scores of practices among the study participants in the control and experimental groups before the intervention.

**Table 12:** Level of Significance for Practices Regarding Safe Home Between the Control Group and Experimental Group in Pretest NS: non-significant

Group	Mean	SD	T-value	P-value
Control	23.38	10.68	1.310	0.193, NS (p>0.05)
Experimental	21.56	9.55		

Table 13 shows the significance level for the difference between the mean scores of practices among the study participants in the control and experimental groups after the intervention.

**Table 13:** Level of Significance for Practices Regarding Safe Home Between the Control Group and Experimental Group in Post-test S: significant

Group	Mean	SD	T-value	P-value
Control	31.14	13.21	16.431	0.000, S (p<0.05)
Experimental	69.40	12.96		

Table 14 shows the significance level for the difference between the mean scores of practices among the study participants in three different observations (one before and two after) in the experimental group.

**Table 14:** Level of Significance for Practices Regarding Safe Home in the Experimental Group S: significant

Test	Mean	SD	T/F-value	P-value
Pretest	21.56	9.55	22.778	0.001, S (p<0.05)
O2	60.67	13.10		
O2	60.67	13.10	7.828	0.001, S (p<0.05)
Post-test	69.40	12.96		
Pretest	21.56	9.55	26.762	0.001, S (p<0.05)
Post-test	69.40	12.96		

Association of study variables with the demographic characteristics of the study participants

Table 15 shows the association of post-test knowledge with the demographic characteristics of the study participants. It reveals that the post-test knowledge scores are not statistically associated with any of the demographic characteristics of the study participants except for ownership of the house (p=0.044, S, as p<0.05). This significant association with homeownership can be attributed to the skewed distribution of participants, with 93% being owners of the house and only 7% residing on a rental basis.

**Table 15:** Association of Post-test Knowledge Scores With Demographic Variables (One-Way ANOVA-Tukey's Test) S: significant; NS: non-significant; ANOVA: analysis of variance; N: number

Demographic Variables	N	Mean	SD	F-value	P-value
Number of children under five					
1	58	26.78	1.60	0.338	0.798, NS (p>0.05)
2	37	26.60	2.32		
3	4	26.50	2.38		
4	1	25.00	-		
Type of house					
Kaccha	20	26.60	1.82	0.044	0.835, NS (p>0.05)
Pakka	80	26.70	1.94		
Ownership of house					
Owned	93	26.79	1.48	4.155	0.044, S (p<0.05)
Rented	7	25.29	4.89		
Type of family					
Nuclear	55	26.44	2.19	2.017	0.159, NS (p>0.05)
Joint	45	26.98	1.45		
Number of family members					
2-4	48	26.35	2.31	1.394	0.249, NS (p>0.05)
5-6	34	26.94	1.28		
7-8	13	27.39	1.61		
More than eight	5	26.20	1.48		
Age of the father					
21-30	24	26.63	2.98	0.020	0.980, NS (p>0.05)
31-40	71	26.70	1.49		
41-50	5	26.60	0.55		
More than 50	0	-	-		
Age of the mother					
21-30	90	26.79	1.91	1.753	0.179, NS (p>0.05)
31-40	9	25.56	1.67		
41-50	1	27.00	-		
More than 50					
Education of the father					
No formal education	0	-	-	0.429	0.827, NS (p>0.05)
Primary	14	26.43	1.40		
Secondary	51	26.90	2.23		
Higher secondary	25	26.48	1.69		
Graduate	5	26.80	1.30		
Postgraduate	4	26.25	0.96		
Others	1	25.00	-		
Education of the mother					
No formal education	0	-	-	1.029	0.405, NS (p>0.05)
Primary	10	26.10	2.23		
Secondary	35	26.89	1.23		
Higher secondary	40	26.95	1.57		
Graduate	12	25.75	3.67		
Postgraduate	2	26.50	0.71		
Others	1	26.00	-		
Occupation of the father					
Unemployed	0	-	-	0.341	0.796, NS (p>0.05)
Self-employed	11	27.18	1.78		
Farmer	25	26.48	2.65		
Service	17	26.65	1.66		
Laborer	47	26.68	1.56		
Occupation of the mother					
Homemaker	82	26.77	1.88	0.519	0.670, NS (p>0.05)
Self-employed	2	26.50	0.71		
Farmer	0	-	-		
Service	3	27.00	2.00		
Laborer	13	26.08	2.22		

Table 16 shows the association of post-test practices with the demographic characteristics of the study participants. It reveals that the post-test practice's scores are not statistically associated with any of the study participants' demographic characteristics except the father's occupation (p=0.027, S, as p<0.05). This significant association with the father's occupation may be attributed to the skewed distribution of participants, with 47% being laborers and 25% being farmers.

**Table 16:** Association of Post-test Practices With Demographic Scores (One-Way ANOVA-Tukey's Test) S: significant; NS: non-significant; ANOVA: analysis of variance; N: number

Demographic Variables	N	Mean	SD	F-value	P-value
Number of children under five					
1	58	67.55	13.92	1.216	0.308, NS (p>0.05)
2	37	71.32	11.72		
3	4	75.75	3.69		
4	1	80.00	-		
Type of house					
Kaccha	20	73.65	10.23	2.736	0.101, NS (p>0.05)
Pakka	80	68.34	13.40		
Ownership of house					
Owned	93	69.98	12.35	2.692	0.104, NS (p>0.05)
Rented	7	61.71	18.89		
Type of family					
Nuclear	55	68.49	13.83	0.599	0.441, NS (p>0.05)
Joint	45	70.51	11.88		
Number of family members					
2-4	48	68.02	14.10	0.389	0.762, NS (p>0.05)
5-6	34	70.29	11.87		
7-8	13	71.85	12.22		
More than eight	5	70.20	12.70		
Age of the father					
21-30	24	72.54	10.40	1.244	0.293, NS (p>0.05)
31-40	71	68.72	13.32		
41-50	5	64.00	18.10		
More than 50	0	-	-		
Age of the mother					
21-30	90	70.14	12.78	2.817	0.065, NS (p>0.05)
31-40	9	60.56	12.18		
41-50	1	82.00	-		
More than 50	0	-	-		
Education of the father					
No formal education	0	-	-	1.061	0.387, NS (p>0.05)
Primary	14	71.00	13.47		
Secondary	51	71.24	12.87		
Higher secondary	25	65.68	12.43		
Graduate	5	68.60	11.70		
Postgraduate	4	62.00	16.81		
Others	1	80.00	-		
Education of the mother					
No formal education	0			1.257	0.289, NS (p>0.05)
Primary	10	72.00	11.29		
Secondary	35	72.11	12.94		
Higher Secondary	40	67.65	13.17		
Graduate	12	66.58	12.50		
Postgraduate	2	55.50	16.26		
Others	1	80.00	-		
Occupation of the father					
Unemployed	0			3.193	0.027, S (p<0.05)
Self-employed	11	75.18	6.59		
Farmer	25	63.56	13.91		
Service	17	67.88	12.28		
Laborer	47	71.70	12.88		
Occupation of the mother					
Homemaker	82	69.90	12.55	0.796	0.499, NS (p>0.05)
Self-employed	2	76.50	4.95		
Farmer	0	-	-		
Service	3	61.00	10.82		
Laborer	13	67.08	16.35		

Table 17 shows the relationship between knowledge and practices in the pretest and post-test. It reveals that pretest knowledge and practices were statistically significantly related (p=0.000). The relationship is strongly positive with r=0.411. Further, it shows that the post-test knowledge and practices were statistically significantly related with a positive correlation coefficient r=0.256 (p=0.010).

**Table 17:** Correlation Between Knowledge and Practices of Safe Home S: significant

Variables	Test	Mean	SD	R	P-value
Knowledge	Pretest	14.96	5.22	0.411	0.000, S (p=0.00)
Practices	Pretest	21.56	9.55		
Knowledge	Post-test	20.11	3.54	0.256	0.010, S (p<0.05)
Practices	Post-test	69.40	12.96		

## Discussion

Compared to other diseases, pediatric injuries get far less attention despite their severity and possibility to be prevented [4]. Each year, more than a million kids under the age of 15 are harmed in incidents around the house in the United Kingdom, for which they are transported to accident and emergency departments. By promoting awareness and making changes to the home environment, most of these events are preventable [5]. A recognized risk factor for all types of harm is caregiver depression. Long-term child health issues, residence, and frequent child care provided by a non-household member are additional risk factors [2].

We focused on studying the domestic injury risk factors and their prevention since doing so would help us create interventions that will alter people's behavior and educate them [6]. For mothers and kids, basic knowledge is necessary, so first aid instruction should begin in kindergarten [7]. According to the current study, mothers had a limited understanding of the four categories of accidents analyzed. This supports a Chinese study's finding that parents' awareness of the prevention of an accident and the promotion of its safety was inadequate [8]. However, these results correlate with a study of 230 Iranian mothers, where it was discovered that 75% had a good understanding [3]. Unexpectedly, none of the mothers' information sources were connected to knowledgeable sources about preventing accidents.

A study that conducted a Middle Eastern survey discovered that the majority of detergents and pharmaceuticals were kept in locked or up-high cabinets [9]. According to a study conducted in Porto Alegre, Southern Brazil, storing dangerous substances below 150 cm increases the risk of poisoning in children by almost 17 times when compared to the control group [1]. Only 20% of the caregivers in a US study of 76 individuals to determine their practices in preventing poisoning at home were found to be aware of the poison control center's phone number [10].

Our findings also showed that there is a lack of awareness regarding how to keep kids away from fire. The greatest risk of home fires is among children under the age of 12. The majority of burns occur in the home from hot water and food-related accidents, whereas direct flame burns are more common as people age [11]. One of the biggest risks to kids is house fires. Children who play with matches and lighters in particular regularly ignite house fires [5].

This study also revealed a lack of the awareness of preventative measures against electrical injury in children. This was in opposition to a 1996 Canadian study that revealed that 63% of mothers protect their kids from electrical device harm [12]. The range of electrical harm includes minor damage, serious multiple-organ involvement, and death. The majority of child injuries occur in the home environment [13]. The Victorian Emergency Minimum Dataset (VEMD) statistics on electrocutions from power points among children aged 0-9 years suggest that more than 70% of incidents occur in this age range [2].

The kitchen is regarded as the most harmful area for kids [14]. A recent study showed that mothers lacked a clear understanding of how to protect their children away from sharp kitchen equipment. This is in opposition to a study by Black in New York, which discovered that 74% of mothers are knowledgeable on how to safeguard their kids from the dangers of knives and other sharp objects in the kitchen [15].

It was shown that mothers' awareness of injury prevention was inversely correlated with the number of years of school; this is quite curious and may be explained by the fact that females with more education are typically employed and away from the home for a significant portion of the day. Children are more likely to have accidents as a result, and mothers may lose interest in or become too busy to educate about preventative techniques. This is in line with a study conducted in Iran, which revealed that mothers' jobs, high school education, and being away from the house for at least eight hours a day were associated with inadequate knowledge and attitudes about preventing domestic injuries [3]. However, research from Egypt found the opposite, demonstrating that the knowledge of preventing child accidents is statistically improved with higher levels of schooling [16], and according to a Canadian study, education level is inversely correlated with the awareness of how to prevent incidents brought on by household chemicals [17].

Limitation of the study

This study was limited to parents of under-five children from selected communities. Those who could not participate may have different levels of awareness regarding preventing domestic accidents. The study was limited only to six months. Although the intervention suggested some architectural changes to make the homes safer for children under five years, most participants could not implement these changes. It was found that most of the participants had their own houses, which they resisted change. The Indian culture does not accept frequent changes in the structure of a home, as most houses are constructed using Vaastu science. However, the minor modifications advised were implemented by the parents. The changes other than the structure of a safe house, as suggested in safe home practices, were readily accepted and implemented by parents. The domain of home safety is restricted to parents alone. Multidisciplinary actions and interventions are required to make children safe at home. The investigator did not control these disciplines as the study was conceived to create awareness among parents of under-five children.

## Conclusions

It can be concluded from this study that the safe home toolkit for under-five children (SHT-UFC) was effective for the awareness of parents regarding the prevention of domestic accidents. Many unusual accidents occur in the home because of the lack of knowledge and preventive practice. Safe home tool kits are helpful in preventing home accidents among children, and they promote a safe environment for children.
